# An open B-mode ultrasound database for deep learning-based atherosclerotic plaque segmentation

**DOI:** 10.1038/s41597-026-06952-7

**Published:** 2026-03-17

**Authors:** Valeria S. Rulloni, Hernan A. Perez, Trinidad Dori, Nahuel A. Borchi, Francisco N. Gil, Bruna de Vargas Guterres, Nestor H. Garcia

**Affiliations:** 1 Facultad de Ciencias Exactas, Físicas y Naturales, Universidad Nacional de Córdoba, Córdoba, Argentina; 2 Facultad de Ciencias de la Salud, Universidad Católica de Córdoba, Libertad 1255, X5004 Córdoba, Argentina; 3 Posgrado en Robótica e Inteligencia Artificial, Universidad Tecnológica del Uruguay, Rivera, Uruguay; 4 Instituto de Investigaciones en Ciencias de la Salud, CONICET, Córdoba, Argentina

## Abstract

Cardiovascular events, predominantly ischemic, account for approximately 32% of global mortality and are expected to increase approximately 30% by 2030. A substantial proportion of these events is preventable through the control of established risk factors. Atherosclerosis is defined by the progressive development of arterial plaques and remains the main cause of ischemic cardiovascular disease. In this context, plaque detection in medical images is required for early diagnosis and longitudinal assessment, which is commonly based on total plaque area. Manual plaque annotation in B-mode ultrasound images requires specialized expertise and is affected by inter- and intra-operator variability. Automatic methods trained on expert-annotated data are therefore required to improve standardization and reproducibility. However, the research community faces a limited availability of open access databases designed for image segmentation and benchmarking under traditional and heterogeneous imaging conditions inherent to B mode ultrasound data. This work presents an open-access B-mode ultrasound image database designed for atherosclerotic plaque segmentation. The dataset includes 541 ultrasound images with a resolution of 800 × 800 pixels, each paired with a binary segmentation mask validated by clinical specialists. The database captures heterogeneous imaging conditions, including plaque-free cases, single and multiple plaques, and non-centralized plaque locations. To assess its suitability for algorithm development, the dataset was used to train a U-Net ensemble. The evaluation provided a median error of 0.35 mm^2^ on plaque free test images and a mean Dice coefficient of 0.62 on test images containing plaques.

## Background & Summary

Atherosclerosis is a pathological condition characterized by lipid deposits in the arterial walls, leading to inflammation and progressive arterial narrowing^1^. It is a significant cause of morbidity and mortality in Western societies due to unhealthy lifestyles^2^. Atherosclerosis generally refers to the hardening of the medium and large arteries^3^. Plaque build-up in the carotid artery can restrict cerebral blood flow and predispose individuals to cerebrovascular events such as strokes^4^.

The Total Carotid Plaque Area (TPA) measurement sums the plaque areas along the carotid artery. It has become a strong predictor of cardiovascular events such as myocardial infarction or stroke^5–8^. Despite its clinical relevance and cost effectiveness, TPA measurement requires the segmentation of atherosclerotic plaques. This task demands extensive training and is therefore limited in routine practice due to potential errors^9^, high workload, and physician fatigue. U-Net neural networks, specialized in medical image segmentation, offer improved accuracy in identifying atherosclerotic plaques and could provide an accessible and consistent tool for predicting cardiovascular events^10^. In this context, the present work describes the process for obtaining an image database suitable for training neural networks to segment atherosclerotic plaques.

Several methods based on traditional image processing and machine learning techniques have been proposed for carotid ultrasound image segmentation. Initial methods, such as those developed by Loizou^11^, were semiautomatic and required manual interaction, making them sensitive to user experience. Qian and Yang^12^ presented a fully automated method for segmenting carotid atherosclerotic plaques, but showed limited consistency in segmenting adjacent pixels, probably due to the use of classifiers that did not take advantage of spatial context.

Current developments include the presence of atherosclerotic plaques and address binary classification at the pixel level, plaque detection using bounding boxes, or image classification into high- or low-risk categories. The researchers Liapi *et al*.^13^ evaluated the impact of preprocessing on the performance of the Channel-wise Feature Pyramid Network for Medicine model for segmenting atherosclerotic plaques. It achieved an improvement of 2–3% in the Dice Similarity Coefficient (DSC) when resolution- and intensity-normalization and despeckling were added. Singh *et al*.^14^ investigated texture characteristics and deep learning on 266 images. Omarov *et al*.^15^ employed the You Only Look Once (YOLO) v8 model for plaque detection using 608 training images and 103 testing images. They reported high accuracy for stroke risk prediction.

While these studies classified or detected plaques, the present database focuses on segmentation. In this context, Huang *et al*.^16^ reviewed deep learning segmentation algorithms, highlighting the previous contributions of Jain *et al*.^17^ and Jain *et al*.^18^. Specifically, Jain *et al*.^17^ compared UNet and SegNet models using Cross-Entropy (CE) and DSC loss functions. The CE-loss models performed better, achieving Jaccard scores between 75% and 82%). A series of studies from the same research group reported results for U-Net-based methods in this application (see also^19,20^). Jain *et al*.^18^ evaluated generalization performance across different ethnic groups using a private multi-ethnic database. It demonstrated that training on a diverse database can support more robust segmentation.

Deep Learning models based on Convolutional Neural Networks (CNNs) have shown promising performance for automatic medical image segmentation. Li *et al*.^21^ employed modified U-Net architectures for intravascular ultrasound images, and Jain *et al*^17^ investigated both hybrid and pure deep learning models for atherosclerotic plaque segmentation in carotid ultrasound images. Liapi *et al*.^13^ also presented a U-Net-based solution for delineating atherosclerotic plaques. Meshram *et al*.^22^, used U-Net and dilated U-Net to segment atherosclerotic plaques, achieving a Dice coefficient of 0.55.

In Latin America, Miyagawa *et al*.^23^ employed CNNs for lumen segmentation in optical coherence tomography images, achieving improved performance at lower resolutions (192×192 pixels). Ensemble neural networks have become increasingly relevant for medical image segmentation. Thambawita *et al*.^24^ combined outputs from architectures such as UNet++, FPN, DeepLabv3, DeepLabv3+, and TriUNet, creating more generalizable segmentation masks. The researchers Zhou *et al*.^25^ employed two private databases (SPARC: 510 ultrasound images from 144 subjects; Zhongnan Hospital: 638 plaque images from 497 subjects) for building an ensemble DL model. The images were manually cropped around each plaque and resized to 96×144, simplifying the task. However, it excluded images without plaques, with multiple plaques, or with non-centered plaques. The proposed solution was built on integrating UNet++ with VGG, ResNet, and DenseNet backbones to achieve more accurate segmentation. Training results showed Dice coefficients of 83.3 ± 10, 85.3 ± 8.3, and 85 ± 7.8 for three repetitions. Testing on Zhongnan images yielded a Dice coefficient of 88.6 ± 6.1.

Although the aforementioned studies have made valuable contributions, several limitations restrict their generalizability to real-world clinical scenarios. High-performance metrics are often achieved under constrained conditions, such as using only images with clearly visible, centrally located single plaques, manual cropping, or excluding negative cases (images without plaques), potentially inflating evaluation metrics. Furthermore, since these databases are often proprietary and not publicly accessible, the broader scientific community cannot reproduce the reported results.

**Fig. 1:**
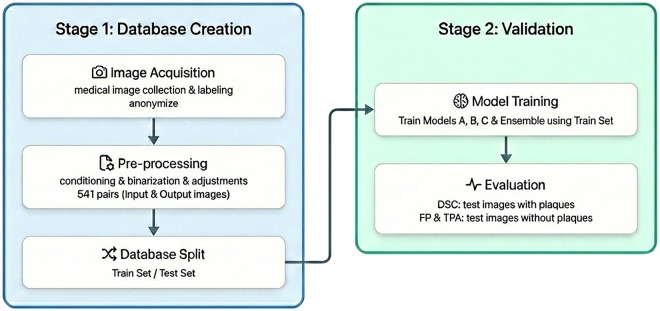
Workflow diagram.

In contrast, the open-access database presented in this work seeks to address these limitations by tackling a broader and more clinically realistic spectrum of data. The workflow, encompassing the end-to-end creation and validation of this resource, is delineated into two primary stages outlined in Fig. 1: Database creation (Stage 1) and Validation (Stage 2). The database creation stage has three important parts:

- Image acquisition
- Image pre-processing and conditioning
- Database split
  The result of this stage is a database containing 541 image pairs (input and output) split into two folders: train and test, suitable for training segmentation algorithms, especially those based on deep learning. The database validation stage has two important parts:

- Model training using the train set
- Evaluation of results using the test set, discriminating analysis according to whether the images have some atherosclerotic plaque or not
  In the validation stage, the models are first trained to produce four segmentation models: Models A, B, C, and an ensemble of them. The performance of these models is then evaluated and compared using the test set, with the analysis distinguishing between images that contain atherosclerotic plaques and those that do not. For images with plaques, the Dice coefficient was used and for images without plaques, the count of pixels predicted as false positives (FP) was used. The total plaque area (TPA) is also shown, scaled by resolution. After these two stages, an adequate validated database is obtained for training predictive algorithms to segment atherosclerotic plaques.

## Methods

The following subsections present the image acquisition protocol and the methodology for image pre-processing for the database construction. Pre processing was performed in Python 3.10 using standard scientific computing libraries, including Numpy 1.26.0, OpenCV 4.6.0, Simple itk 2.2.1, and Pydicom 2.4 in the Github environment.

### Image acquisition

The image acquisition and clinical evaluation protocol was approved by the Independent Institutional Ethics Committee of Health Research of the Universidad Nacional Córdoba (CIEIS HNC-FCM Protocol N^°^: 119/12, June 28, 2012) and the Rusculleda Foundation (September 26, 2013). Each participant provided written informed consent regarding treatment options and data sharing.

**Fig. 2:**
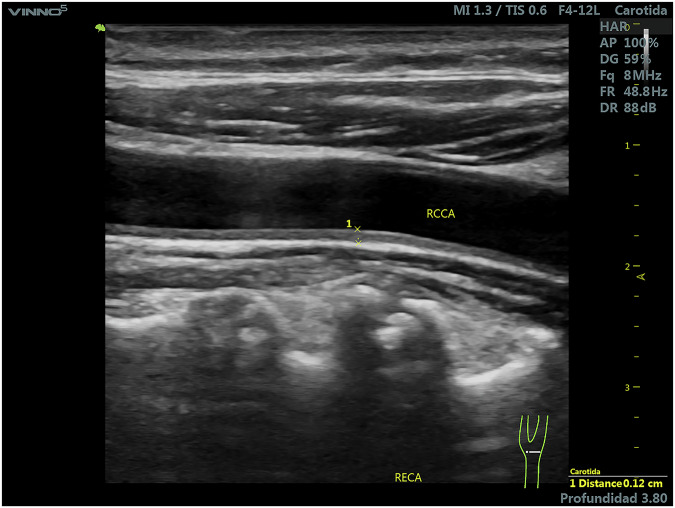
Raw image example.

**Fig. 3:**
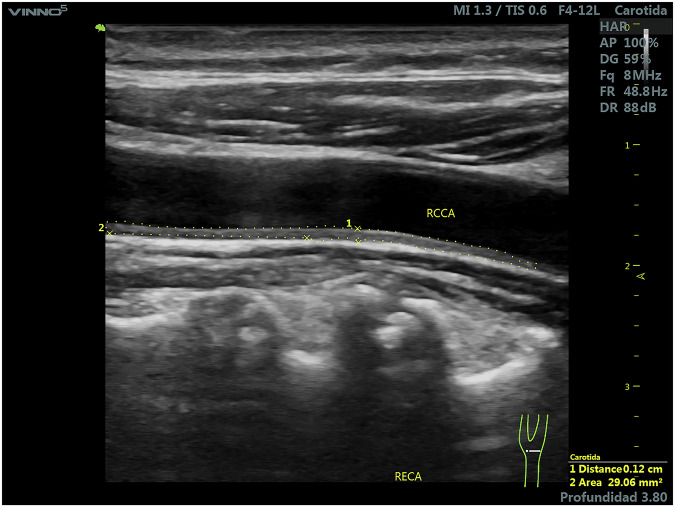
Marked image example.

Although the data and image collection phase of the project met the initial goals of the TPA study, as documented in articles^9,26^, the characteristics of the initial images were not suitable for machine learning algorithms in binary segmentation. Then ultrasound images were obtained from a high-risk cohort (57% male) in a cardiovascular prevention program (at the Blossom DMO Private Center in Cordoba, Argentina), extended through September 2023. While initial clinical characteristics for the full cohort are published by Pérez *et al*.^27^ (and summarized in Table 1), a rigorous image screening process was applied for this study to ensure images were suitable for machine learning. Since our primary objective is to develop a tool that replicates expert human plaque segmentation, we focused on image quality over individualized sub-cohort epidemiology. These tests were part of a grant and collaboration agreement between the Sadosky Foundation and Eira Company. The trials involved acquiring longitudinal B-mode ultrasound images of the cervical region, from the base of the neck to the carotid bifurcation. The study included male and female patients of different ages with mild, moderate, or severe atherosclerosis. Inclusion criteria comprised ultrasound images exhibiting either the absence or presence of arterosclerotic plaques, where the latter was defined as having a wall thickness greater than 1 mm, in accordance with Spence *et al*.^5^ The professionals provided images identified as free of plaques and images containing one or more plaques. In the latter cases, each image was accompanied by one or two corresponding reference images, in which the plaques were manually delineated by the physicians. All images were anonymized. Throughout this work, *raw images* refer to ultrasound monochrome images that containg none, one or more atherosclerotic plaques without demarcation, while *marked images* are replicas of the former, in which the perimeter of the plaque has been manually annotated, as seen in Figs. 2 and 3.

**Table 1:** Summarized clinical and demographic characteristics of the high-risk cohort.

Category	Parameter	Summary / Value
**Demographics & Vitals**	Gender	57% Male, 43% Female
	Age	Middle-aged to elderly
	Blood Pressure	35% hypertensive (Mean SBP: ~124, DBP: ~72 mmHg)
**Biochemical Profile**	Lipids	27% hypercholesterolemia (Dyslipidemia up to 84.6%)
	Glucose	14% Diabetes. Mean HbA1c: 5.8–6.1%
	Renal Function	eGFR ranging from 20 to 100
**Risk Categorization**	Framingham Score	> 15% (subclinical atherosclerosis)
	Clinical Context	Primary & secondary prevention

Ultrasound images were acquired using two different devices from the Esaote and Vinno manufacturers. The image format was defined by the specific capabilities of these ultrasound machines: from these acquisitions, a total of 26 pairs of useful images in DICOM format and 515 pairs of images in PNG format were obtained, respectively. This resulted in an initial original database containing 541 pairs of images (*raw* and *marked*). DICOM images provided a spatial resolution of 0.106 mm per pixel and an image size of 800 × 600 pixels. In contrast, the PNG images were taken at varying depths (3.5 to 5 cm), resulting in pixel sizes of 0.04 to 0.06 mm and an original image size of 1200×900 pixels. Within the resulting data set, 201 pairs of images correspond to cases with no plaque, along with 340 pairs showing the presence of some atherosclerotic plaque. This inclusion of images acquired at different scales and resolutions contributes to the database’s heterogeneity, potentially supporting the model’s ability to generalize across varying imaging conditions and acquisition protocols.

### Image Pre-Processing

The resulting database incorporated a variety of artifacts and characteristics inherited from the image annotation protocol and the specific ultrasound devices used during acquisition. In this sense, image preprocessing plays an important role in producing a clean, standardized dataset suitable for further processing and model training. Image artifacts include crosses, marks, acronyms, numbers, and descriptions of the clinical examination. These elements may appear either within or outside the region of interest in the monochrome ultrasound image. It is also worth mentioning that the plaques were delineated through yellow dots and green stripes for the PNG and DICOM images, respectively. In order to address these issues and ensure a consistent representation of the images, a semi-automatic preprocessing pipeline was developed, consisting of the following steps:

- **Image Assessment:** Assess the characteristics of each image or pair of images.
- **Artifact Identification:** Identify each artifact and its location using metadata and template matching.
- **Artifact Removal:** Remove identified artifacts through pixel replacement strategies, leveraging surrounding non-affected pixels (a 5×5 neighborhood) to preserve local image consistency.
- **Standardization and Cropping:** Crop all images to a standardized size of 800 × 800 pixels, ensuring the relevant echographic image zone is preserved. Zero-padding was applied to smaller images to reach the required size.
- **Plaque Segmentation:** Compare raw and marked images to create a binary image with white pixels within the plaques. For this segmentation, we subtract the RGB layers (*G* − *B*), binarize the result, apply morphological closing to delineate plaque boundaries, and then perform hole filling.
- **Filling Correction:** Correct errors in the filling process that arose from points being too close or too far apart.

**Fig. 4:**
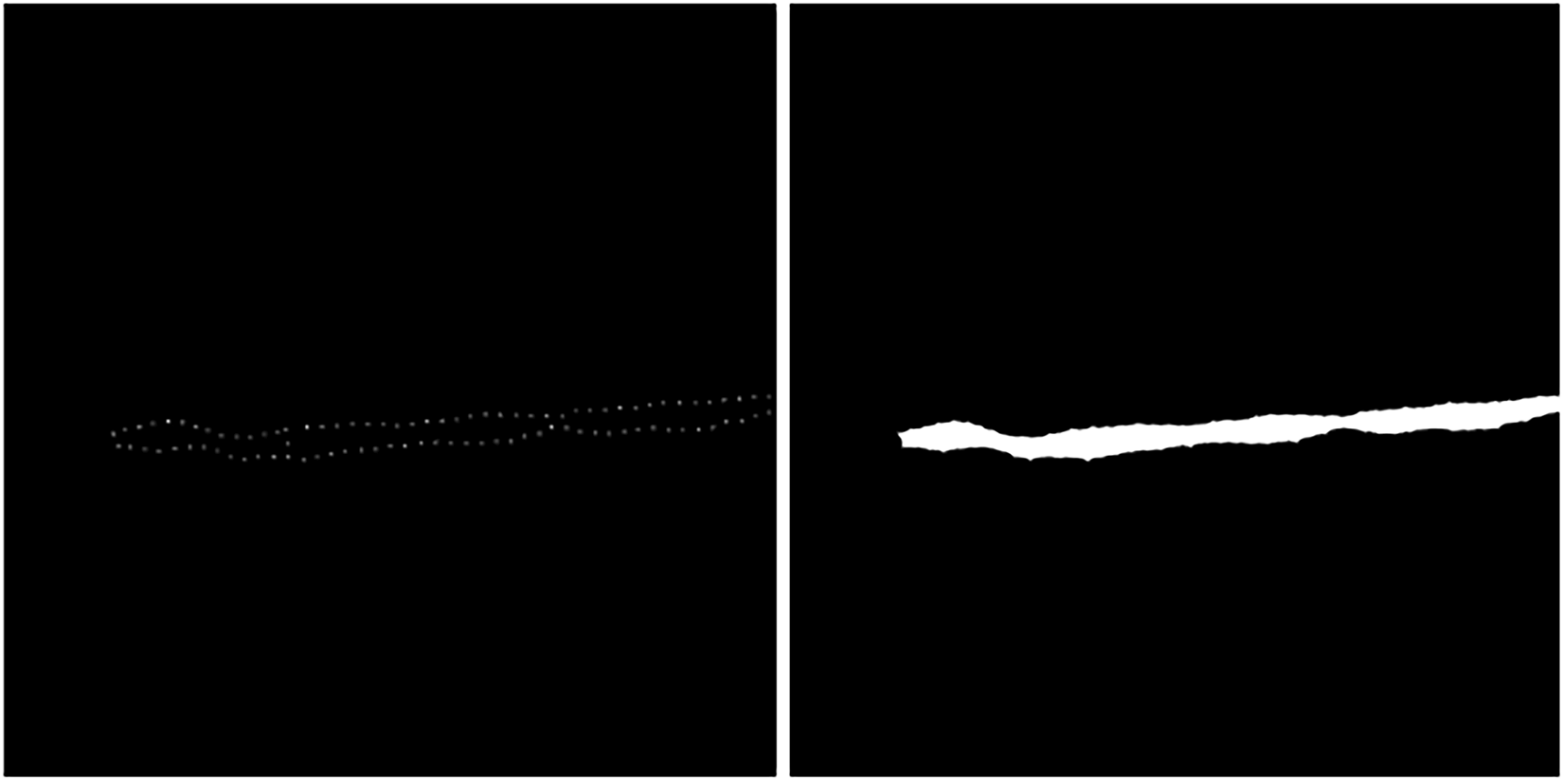
An example of step 5: on the left, the first binarization after we subtract RGB layers (G-B) with dots, and in the right, the result after filling the hole.

**Fig. 5:**
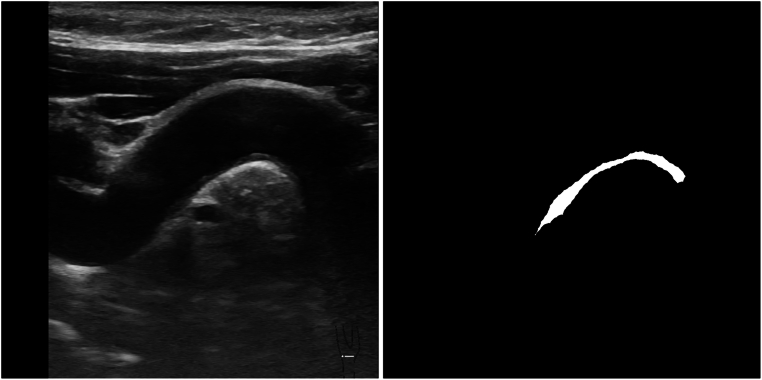
A monochrome input image and its binary output image, both 800 × 800.

The most important functions of the opencv library (cv2) used in this section were: *matchtemplate, findcontours, drawcontours, minMaxLoc, rectangle*, and *threshold*. In step 2, artifacts are identified using metadata and template matching, similar to the approach taken by Mora, Soares, and Fonseca^28^. Step 2 first treats the artifacts as missing data and then imputes those values using the average of the closest valid neighbors. Figure 4 shows an example of the first binarization after subtracting RGB layers (G-B) and the result after filling the hole in step 5. The proposed pre-processing pipeline outputs, for each monochrome image, a pair of 800 × 800 pixel images: a monochrome ultrasound image serving as the model input, and a corresponding binary mask image, in which the plaque regions are represented by white pixels. Figure 5 illustrates an example of the resulting image pair. To ensure format uniformity, all images were saved in uncompressed PNG format, aligning with the output of the Vinno system. This resulted in a database containing 541 pairs of large images (800 × 800). Each image pair consists of one monochrome ultrasound image and its corresponding binary mask.

## Data Records

It is worth noting that the present database provides open-access data in accordance with the FAIR (Findable, Accessible, Interoperable, Reusable) principles. It has been released under the Creative Commons 4.0 International license (CC BY) through the Mendelay Data repository at the following link: 10.17632/8srkpz52dy.2^29^.

To achieve an adequate basis for machine learning, the images achieved in the previous subsection were separated into two sets, one for training and one for testing. Of the 541 pairs of images obtained after pre-processing, 48 pairs were selected for the testing set (9%) using stratified random sampling, with 24 images without plaque and 24 with plaque, leaving the remaining 493 for the train set (91%). The resulting image database is stored in the ‘data’ folder, which contains two sub-folders: ‘test’ and ‘train’. The ‘test’ folder holds the images designated for algorithm evaluation, and the ‘train’ folder holds the images designated for algorithm training. In both sub-folders, the files consist of pairs of PNG images, identified by the index *i*. Each pair consists of a monochrome image named ‘i.png’ and a binary image of the same size containing the segmentation of plaque presence in the monochrome image, named ‘i_labeled.png’. The ‘test’ folder contains 48 image pairs, and their indices range from 497 to 545, excluding 514. The ‘train’ folder contains 504 image pairs, with indices ranging from 1 to 498 and from 546 to 552, and 10 missing indices.

## Technical Validation

Technical Validation was performed within a Python 3.10 environment using Google Colab Pro++, and the most important libraries used were numpy 1.23.5, opencv-python 4.7.0.72, pandas 2.1.4 and tensorflow 2.12.0 (h5py 3.8.0). To evaluate the database, we developed and used an automatic competing algorithm for segmentation: an alternative version of the U-Net++ ensemble model that uses voting and transfer learning. This approach integrates three U-Net sub-networks: VGG16^30^, ResNet50, and ResNet152^31^, named A, B and C respectively, whose encoders were initialized with weights pre-trained on ImageNet database and subsequently frozen to serve as fixed feature extractors. Images from the database intended for training were used to train the models. The images intended for testing from the database were used to evaluate de performances of the models.

Specifically, model training employed three kinds of data augmentation on the 493 pairs of train images using vertical and/or horizontal flips and translations (cropped to 512×512 images) from the center in both directions in multiples of 32 pixels:

- **Model A:** U-Net+VGG16 model trained for 10 epochs using 3940 (3940 = 493×2×4) images. The 3940 images were generated from an initial set of 493 images, augmented through 2 translations by multiples of 32 pixels horizontally (*i* ∈ {0}) and vertically (*j* ∈ { − 2, 2}), and 4 different flip configurations (none, vertical, horizontal, and both).
- **Model B:** U-Net+ResNet50 trained for 16/100 epochs with 9860 (9860 = 493×5×4) images. These images were generated from an initial set of 493 images, augmented through 5 translations by multiples of 32 pixels horizontally (*i* ∈ {0}) and vertically (*j* ∈ { − 4, − 2, 0, 2, 4}) and 4 different flip configurations (none, vertical, horizontal, and both).
- **Model C:** U-Net+ResNet152 trained for 24/40 epochs with 11832 (11832 = 9860 = 493×6×4) images. These images were generated from an initial set of 493 images, augmented through 6 translations by multiples of 32 pixels horizontally (*i* ∈ { − 4, 4}) and vertically (*j* ∈ { − 4, 0, 4}), and 4 different flip configurations (none, vertical, horizontal, and both).

The early stopping criterion was used for model training and the following specifications were used: *Loss* = 1 − *DSC* loss function, ADAM optimizer, learning rate *η* = 0, 0001, validation split=0.2 and a batch size of 16. For both the calculation of the loss function and the evaluation of the models, the calculation of the Dice Similarity Coefficient (DSC) used the counts of false positives (FP), false negatives (FN), true positives (TP), and a smoothing factor *ϵ* = 1*e* − 5 to avoid division by zero as follows:

1 $$DSC=\frac{2TP+\epsilon }{2TP+FP+FN+\epsilon }$$

The ensemble model construction strategy based on Models A, B, and C integrates individual models using a pixel-by-pixel voting-based ensemble function, in which a plaque is predicted only when it is identified by at least two of the three models.

The performance of the models was analyzed using the test set, accounting for the different image characteristics in the database. For the images containing plaques, segmentation performance was assessed using the Dice Similarity Coefficient. The mean of the Similarity Coefficient for models A, B, C, and their ensemble was 0.57, 0.57, 0.6, and 0.62, respectively. A comparative table including the mean and standard deviation of each model can be seen in Table 2. For the plaque-free images, the evaluation focused on false positive (in pixels) predictions. The median false-positive count for models A, B, C, and their ensemble was 1153, 0, 673, and 96, respectively. A comparative table with statistical measures of each model can be seen in Table 3.

**Table 2:** Dice Similarity Coefficient (DSC) Mean and Standard deviation results by model.

Model	DSC
A (VGG16)	0.57 ± 0.2
B (Resnet50)	0.57 ± 0.2
C (Resnet152)	0.6 ± 0.2
Ensamble	0.62 ± 0.23

**Table 3:** False Positive metrics for each model.

Model	Mean	Variance	P25	Median	P75
A (VGG16)	1465	1623	31	1153	2295
B (Resnet50)	501	843	0	0	652
C (Resnet152)	1466	1727	1	673	2541
Ensamble	686	915	0	96	1178

**Fig. 6:**
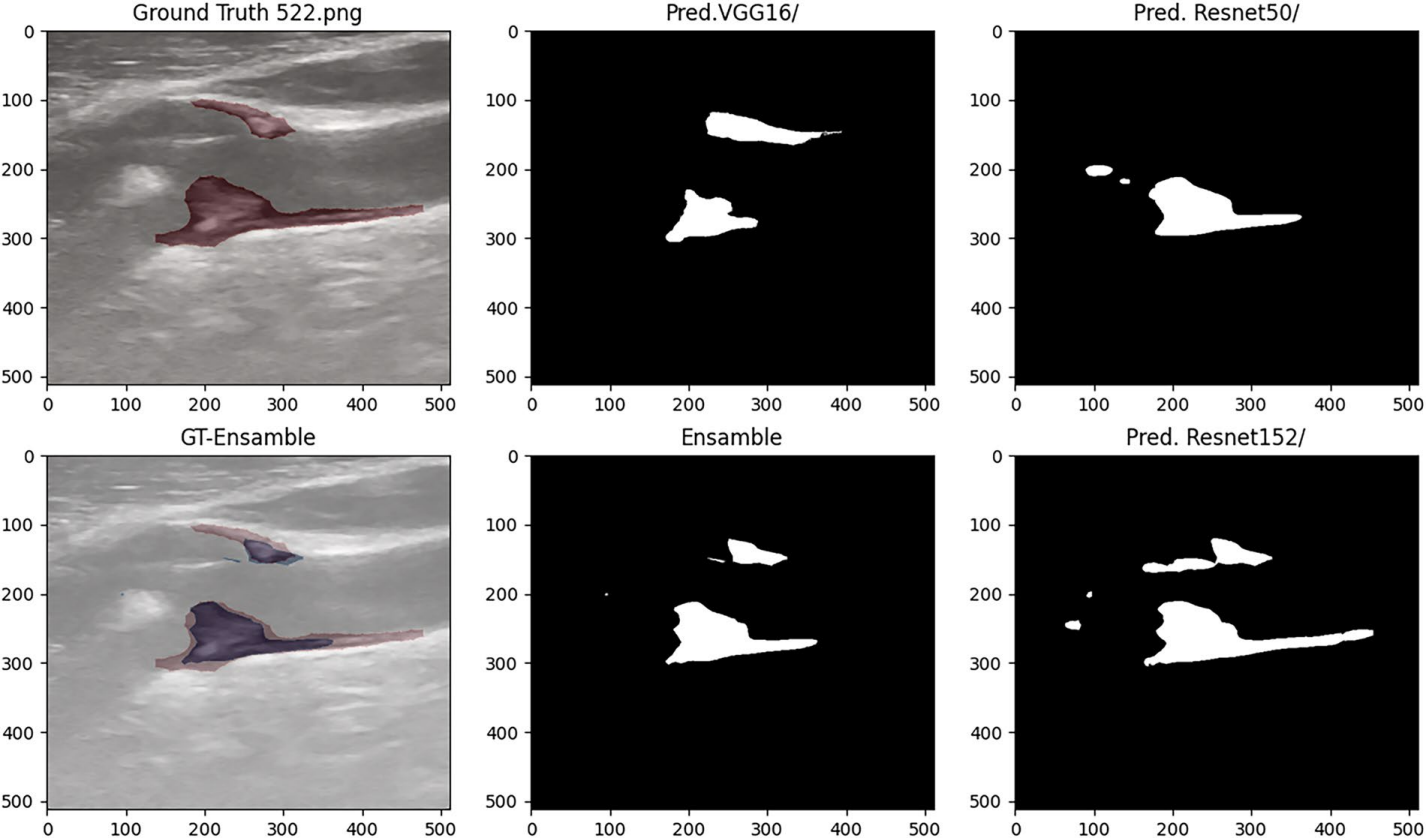
Test Image Showing Ground Truth and Predictions (DSC: 0.4959 Mod A, 0.6548 Mod B, 0.7612 Mod C, and 0.7203 Ensemble).

**Fig. 7:**
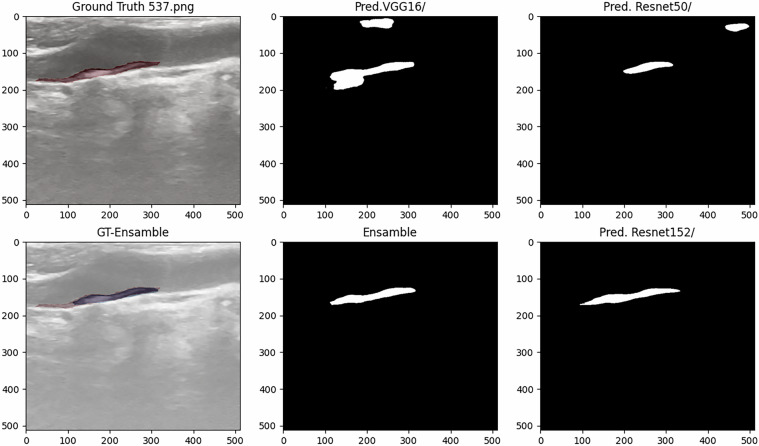
Test Image Showing Ground Truth and Predictions (DSC: 0.5746 Mod A, 0.4544 Mod B, 0.7605 Mod C, and 0.7828 Ensamble).

**Fig. 8:**
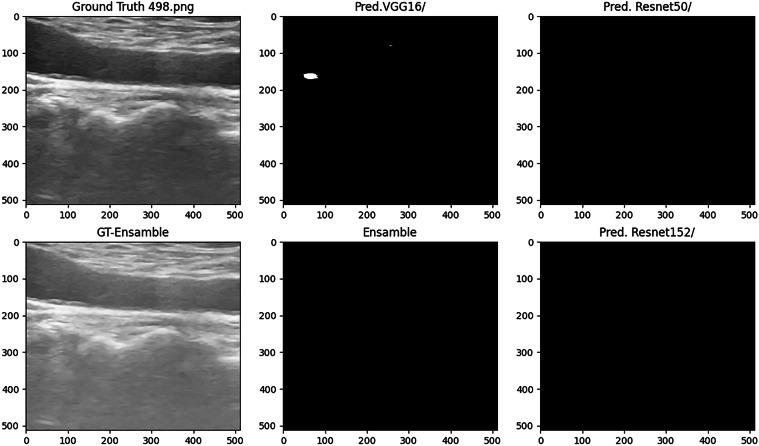
Test Image Showing Ground Truth (without plaque) and Predictions.

**Fig. 9:**
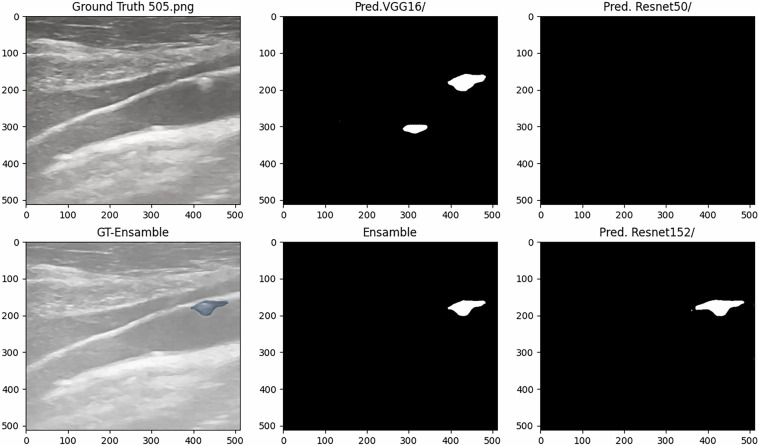
Test Image Showing Ground Truth (without plaque) and Predictions (Ensamble’s False Positives in blue).

Figures 6 and 7 present the predictive results of models A, B, C, and their ensemble for two testing images with plaques, while Figures 8 and 9 provide examples of the model’s predictions in testing plaque-free images.

Considering that one of the most important objectives of plaque segmentation is the posterior calculation of total plaque area, it should be noted that once the algorithm has predicted the segmentation, the calculation is very simple. Simply multiply the number of pixels belonging to all the segmented plaques by the pixel area given by the spatial resolution (*TP* + *FN*)*pixelspacing^2^, which in our case varies between 0.04^2^ and 0.06^2^ for images from the Vinno ultrasound machine and 0.106^2^ for images from the Esaote ultrasound machine. Considering that the generated database no longer has the specific resolution of each image, it is not possible to calculate the total predicted plaque area. However, by using the results of the images without atherosclerotic plaques and multiplying them by 0.06^2^, we can make an estimated calculation of the errors for the images without plaques: The estimated median of the TPA for models A, B, C, and their ensemble were 4.15, 0, 2.42, and 0.35 *mm*^2^, respectively.

## Discussion

Based on the analysis of quantitative metrics and qualitative visualizations (Figs. 6, 7, 8, and 9), the following points can be highlighted:

- The ensemble model demonstrates the ability to compensate for the limitations of individual models and to equilibrate the diversity of the database by eliminating individual false positives from each model when there are none in the other models. This situation can be visualized in Figs. 6, 7, 8, and 9). This translates into more stable predictions and robustness across the proposed heterogeneous imaging conditions.
- The medical analysis carried out by the doctors supported the competent performance of the computational results. This competence approaches an acceptable level and is comparable to the inter-observer variability inherent to the problem, as well as to the work of Zhou *et al*.^25^, which served as inspiration for this study.

In the research of Zhou *et al*.^25^, with a larger but private database, data augmentation strategies, and an ensemble of 8 networks, a Dice coefficient of 83.3–85.7% was achieved. Crucially, when contrasting our results with those work, we must consider some differences between the seminal study and our current database study:

- The aforementioned study used manually selected 96 × 144 pixel images focused on a plaque area, requiring operator intervention. Our method uses a more comprehensive approach with 512×512 pixel images, covering almost the entire raw image without manual selection of relevant regions.
- During the training of the algorithms proposed in Jain *et al*.^19,20 and Zhou *et al*.25 ^, images without plaques as well as those containing more than one plaque per image were excluded. This represents a significant difference compared to our work, algorithms, and database, which consider image segmentation independently, whether it contains one, multiple plaques, or none at all.
- In contrast to these studies, which rely on proprietary datasets, our database is publicly available for research use, along with the source code to facilitate validation and reproducibility.

Training segmentation algorithms and evaluation using plaque-free images presented challenges and particularities such as:

- Standard metrics like DSC and Jaccard are limited when the Ground Truth is empty. To address this, a smoothing parameter was included in training. This prevented division by zero in the loss function denominator and was applied to the numerator, allowing loss function minimization even with an empty Ground Truth by focusing on reducing erroneous predictions.
- False positives were chosen as a more appropriate measure for these instances, as they represent the only detectable errors when no plaque is present.

## Usage Notes

Although this work aimed to include previously unconsidered situations (e.g., the absence of plaques) and to cover a broader image area, the algorithm has a bias due to the specific population and geographical location it represents. This database (with 800 × 800 pixels images) is expected to be a key component in a larger ensemble of databases and models for a comprehensive professional assistance system. It is suggested to augment the database with images from different countries that adhere to the following protocol: pairs of images, each 800 × 800 pixels or smaller, with one image monochrome and the other binary, and with the segmentation of the atherosclerotic plaque(s) present or absent in the image. This should include images with a single atherosclerotic plaque, multiple plaques, and images without any plaques. The databases with smaller-sized images, such as those provided by Zhou *et al*.^25^ could also be integrated, creating 512 × 512 or 800 × 800 pixels images containing the images or subimages with black backgrounds. We encourage researchers to contribute to enriching the database and models, especially by incorporating scenarios beyond the scope of this study.

The present work successfully built an open-access image database tailored for medical applications in atherosclerotic plaque analysis. It yielded interesting results in segmenting atherosclerotic plaques, informed by medical expert insights and feedback. The evaluation code is also accessible at the same location as the database to ensure straightforward reproducibility. The ensemble of UNet-based models effectively combined the limited understanding of each individual model to achieve a more accurate collective segmentation. The results also highlighted significant drawbacks in current approaches, fostering valuable discussions. Typically, current research focuses on analyzing atherosclerotic plaques from the perspective of patients who need them segmented. However, considering scenarios in which images do not show any plaques or contain multiple plaques in a single image is crucial for accurate patient diagnosis. The results suggest that addressing these cases presents additional challenges and indicate that collaborative models may pave the way forward to meet this need.

Furthermore, it is essential to remember that the results derived from plaque-free images must be evaluated differently from those from images containing plaques, as performed in the Technical Validation section.

## Data Availability

The dataset used in this study is publicly available and was released under the Creative Commons Attribution 4.0 International (CC BY) license, in accordance with the FAIR principles. The database is hosted in the Mendeley Data repository: ‘Valeria Rulloni, Hernán Pérez, Trinidad Dori, Nahuel Borchi, Francisco Gil, Bruna de Vargas, Néstor García. Ar-PlaqSegm1: First Argentine database of B-mode ultrasound images for atherosclerotic plaque segmentation’, 10.17632/8srkpz52dy.2^29^.

## Code availability

The code used to train and evaluate the models on this dataset, with results reported in the Technical Validation section, is available at the following link: 10.24433/CO.6142529.v1^32^.
